# Insights into Catalytic and tRNA Recognition Mechanism of the Dual-Specific tRNA Methyltransferase from *Thermococcus kodakarensis*

**DOI:** 10.3390/genes10020100

**Published:** 2019-01-30

**Authors:** Aiswarya Krishnamohan, Samantha Dodbele, Jane E. Jackman

**Affiliations:** The Ohio State Biochemistry Program, Center for RNA Biology and Department of Chemistry and Biochemistry, The Ohio State University, Columbus, OH 43210, USA; krishnamohan.1@buckeyemail.osu.edu (A.K.); dodbele.1@buckeyemail.osu.edu (S.D.)

**Keywords:** tRNA methylation, enzyme mechanism, Trm10

## Abstract

The tRNA methyltransferase Trm10, conserved throughout Eukarya and Archaea, catalyzes N1-methylation of purine residues at position 9 using *S*-adenosyl methionine as the methyl donor. The Trm10 family exhibits diverse target nucleotide specificity, with some homologs that are obligate m^1^G_9_ or m^1^A_9_-specific enzymes, while others are bifunctional enzymes catalyzing both m^1^G_9_ and m^1^A_9_. This variability is particularly intriguing given different chemical properties of the target N1 atom of guanine and adenine. Here we performed an extensive kinetic and mutational analysis of the m^1^G_9_ and m^1^A_9_-catalyzing Trm10 from *Thermococcus kodakarensis* to gain insight into the active site that facilitates this unique bifunctionality. These results suggest that the rate-determining step for catalysis likely involves a conformational change to correctly position the substrate tRNA in the active site. In this model, kinetic preferences for certain tRNA can be explained by variations in the overall stability of the folded substrate tRNA, consistent with tRNA-specific differences in metal ion dependence. Together, these results provide new insight into the substrate recognition, active site and catalytic mechanism of m^1^G/m^1^A catalyzing bifunctional enzymes.

## 1. Introduction

tRNAs undergo extensive post-transcriptional modifications, with the resulting nucleotide diversity affecting the biological and structural properties of these RNAs. Methylation of nucleotides is the most common post-transcriptional modification that can occur, with at least 13 different methylated nucleotides found in all three domains of life [1,2,3]. Almost all the methyltransferases (MTases) that catalyze these modifications in tRNAs belong to two major structural classes (i) Class I Rossman-fold MTases (RFM), which are characterized by the namesake protein fold and generally function as monomers or higher order oligomers and (ii) SpoU-TrmD or SPOUT MTases, which are characterized by a topological knot in the structure and generally form their active site at a homodimeric interface [3,4,5]. 

Trm10, the tRNA m^1^R_9_ MTase (R = G or A) that catalyzes N1-methylation at position 9 of tRNAs in Eukarya and Archaea, is an atypical member of the SPOUT family that acts in monomeric form [6,7,8,9]. Multiple observations underscore the importance of these enzymes and m^1^R_9_ modification in the cell. First, loss of m^1^A_9_ methylation in mammalian mitochondrial tRNA^Lys^ (catalyzed by a human mitochondrial Trm10 homolog hTRMT10C) results in acute misfolding, forming an extended hairpin loop thus making the tRNA unusable in translation [10]. Second, mutations in two different human paralogs (hTRMT10A and hTRMT10C) have been implicated in multiple disease states [11,12,13,14,15]. Third, yeast strains lacking Trm10 (*trm10Δ*) show severe hypersensitivity to the drug 5-fluorouracil [16]. These observations add Trm10 to the growing list of tRNA-modifying enzymes that have critical cellular functions that remain incompletely understood.

**Figure 1:**
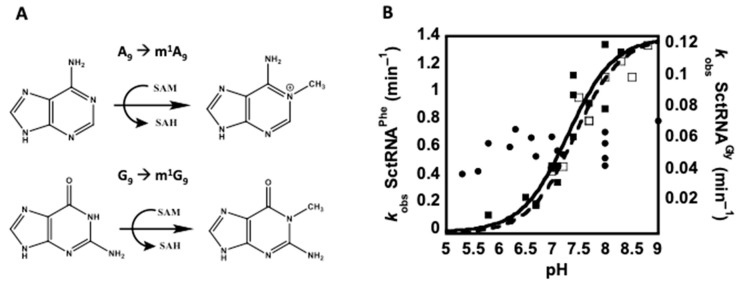
m^1^A_9_ and m^1^G_9_ catalysis by TkTrm10. (**A**) TkTrm10 uses *S*-adenosyl methionine (SAM) as the methyl-group donor for m^1^A_9_ and m^1^G_9_ formation. (**B**) pH-rate profiles for m^1^G_9_ vs m^1^A_9_ formation catalyzed by TkTrm10. Single turnover *k*_obs_ (min^−1^) rates at saturating enzyme concentration were measured as a function of pH for SctRNA^Phe^ (m^1^A_9_, filled circles), SctRNA^Phe^-G9 (m^1^G_9_, filled squares), and SctRNA^Gly^ (m^1^G_9_, open squares). Data from at least three independent experiments were fit to Equation (2) to obtain the reaction pKa values for SctRNA^Phe^-G9 (7.3 ± 0.1) and SctRNA^Gly^ (7.4 ± 0.1), with errors representing the error associated with the curve fit. Note the difference in the *Y*-axis scale for comparison of SctRNA^Phe^ (filled symbols) and SctRNA^Gly^ (open symbols) rates.

Additionally, while all other known N1 MTases exhibit strict specificity for a single target nucleotide base, Trm10 is the only family in which some single enzyme family members display dual specificity, with the ability to methylate both purines [17]. This is a particularly intriguing property given that the N1 atoms of the two purines are characterized by very different pKa values. Guanine N1 is protonated at physiological pH and predictably requires deprotonation before methylation, unlike adenine that lacks the N1 proton at physiological pH (Figure 1A). Adding further complexity, nucleotide substrate specificity varies widely among members of the Trm10 family, and homologs can be either m^1^G_9_-specific (as in *Saccharomyces cerevisiae* (ScTrm10)) [6], m^1^A_9_-specific (as in *Sulfolobus acidocaldarius* (SaTrm10)) [17], or bifunctional m^1^G_9_/m^1^A_9_ (m^1^R_9_) MTases (as in *Thermococcus kodakarensis* (TkTrm10) and the human mitochondrial homolog (hTRMT10C)) [17,18]. The molecular basis for this catalytic flexibility within the Trm10 family remains entirely unknown and thus characterization of the unique active site associated with m^1^R_9_ activity is of significant biochemical interest. An aspartate residue (D245), found to be highly conserved in archaeal enzymes that catalyze m^1^A_9_ activity, but not in any m^1^G_9_-specific enzymes, was proposed to confer some distinctions in the substrate nucleotide [8]. However, this residue is not found in hTRMT10C, another bifunctional enzyme, and mutational analysis of this residue in the context of *T. kodakarensis* Trm10 (TkTrm10) did not result in significantly altered nucleotide specificity [9]. Therefore, enzyme features that confer nucleotide specificity remain to be determined. Comparison of several available crystal structures also failed to provide obvious clues regarding their distinct nucleotide specificities, particularly since these were obtained in the absence of the tRNA substrate [7,8,9]. 

Recent mechanistic characterization of ScTrm10 provided evidence for a distinct catalytic mechanism of m^1^G_9_ modification compared to other SPOUT-dependent MTase enzymes [19]. Although this work answered some questions with respect to m^1^G_9_ catalysis, whether enzymes that catalyze m^1^A_9_ methylation share similar mechanistic features was not addressed. Here, we performed an extensive kinetic analysis of m^1^R_9_ formation by TkTrm10 coupled with a comprehensive mutational analysis of conserved active site residues. These studies suggest a conserved catalytic mechanism for single and dual-specific Trm10 enzymes and provide insight into the molecular basis for discrimination between different tRNA substrates.

## 2. Materials and Methods 

### 2.1. Mutagenesis and Purification of TkTrm10 Variants

Wild-type TkTrm10 was cloned into AVA421, a previously described plasmid for heterologous expression of N-terminal His_6_-tagged protein in *E. coli,* from *T. kodakarensis* genomic DNA using ligation-independent cloning [6,20]. This construct was then used to generate all other TkTrm10 variants through Phusion mutagenesis (Thermo Scientific, Waltham, MA, USA). After verifying the correct sequence through sequencing, the proteins were expressed and purified using metal ion affinity chromatography, as previously described [20] and dialyzed into a storage buffer containing 50% glycerol, 20 mM Tris pH 7.5, 150 mM NaCl, 1 mM MgCl_2_, 1 μM ethylenediamine tetraacetic acid (EDTA), and 0.5 mM dithiothreitol (DTT). After purification, some variants were substantially less pure than the wild-type enzyme, as judged by the fraction of the full-length TkTrm10 protein (44 kDa) band over lower molecular weight species observed in the SDS-PAGE gel (Figure S1). Since the protein concentrations determined for this work by BioRad protein assay (Hercules, CA, USA) reflect total protein concentration, the actual concentration of the low-purity Trm10 variants used in kinetic assays is in some cases lower than the estimated value. However, the single-turnover rates reported in this work were measured at saturating protein concentrations, yielding rates that are independent of the actual protein concentrations (demonstrated by measurement with at least two different enzyme concentrations for each variant) and therefore varied levels of contaminating protein impurities are unlikely to affect the comparisons made between TkTrm10 variants and wild-type enzyme.

### 2.2. In Vitro Transcription and Preparation of tRNA Radio-Labeled Substrates

*T. kodakarensis* native tRNA^Arg^CCG and tRNA^Thr^CGU was cloned from genomic DNA into a plasmid designed for in vitro transcription. Transcripts of these *T. kodakarensis* tRNAs (TktRNA^Arg^ and TktRNA^Thr^) and *S. cerevisiae* tRNAs (SctRNA^Gly^, SctRNA^Phe^ and SctRNA^Phe^-G9 were prepared as previously described [6,20]. The TktRNAs were uniformly labeled by in vitro transcription in the presence of [α-^32^P]GTP or [α-^32^P]ATP [20]. The SctRNAs were specifically labeled with ^32^P at the phosphate immediately 5’ to the A_9_ or G_9_, using methods previously described [20]. 

### 2.3. Single Turnover Kinetics 

Reactions containing the indicated labeled tRNAs, 1.5 mM MgCl_2_, 50 mM Tris pH 8.0, 0.5 mM *S*-adenosyl methionine (SAM) were incubated with ≥2.5 µM enzyme (≥100-fold excess over tRNA concentration) at 40 °C (SctRNAs) or 50 °C (TktRNAs). Aliquots were taken at designated time points and quenched with phenol: chloroform: isoamyl alcohol (25:24:1), purified, digested with Nuclease P1 and analyzed by thin layer chromatography (TLC) in isobutyric acid:H_2_O:NH_4_OH (66:33:1) to resolve G/m^1^G_9_ and saturated ammonium sulfate:H_2_O:isopropanol (80:18:2) to resolve A/m^1^A_9_. Dried TLC plates were exposed to a phosphor screen and scanned using the Typhoon™ imaging system (GE Healthcare, Chicago, IL, USA) and quantified using ImageQuant™ TL software (GE Healthcare). The percent m^1^A_9_/m^1^G_9_ formed (%*P*) was plotted as a function of time (*t*), and *k*_obs_ was determined by fitting to a single exponential equation (Equation (1)) using Kaleidagraph software (Synergy, Reading, PA, USA).

%*P* = *P*_max_ × (1 − exp(−*k*_obs_ × *t*)) (1)

The rates were verified to be saturating for enzyme concentrations by measuring *k*_obs_ with at least two different excess enzyme concentrations for WT, all alanine variants and the 3DE (D100N+D210N+D245N+E111Q) variant. 

### 2.4. pH-Rate Analysis

For the pH-rate profile, single turnover rates were determined as above at various pH conditions using a previously described buffer system [19]. The *k*_obs_ values obtained from two (with SctRNA^Gly^) or three independent experiments (with SctRNA^Phe^ and SctRNA^Phe^-G9) were plotted as a function of pH and the pKa was calculated by fitting the m^1^G_9_ rates to Equation (2) using Kaleidagraph (Synergy), with *k*_max_ representing the pH-independent maximal rate.

*k*_obs_ = *k*_max_/(1 + 10^(pKa−pH)^) (2)

To ensure that all *k*_obs_ values were independent of enzyme concentration, the pH-rate analysis for catalysis of SctRNA^Phe^ and SctRNA^Phe^-G9 was performed using at least two different excess enzyme concentrations, revealing saturated rates at [TkTrm10] ≥ 2 µM. The analysis for SctRNA^Gly^ and the TktRNAs was therefore similarly performed at [TkTrm10] ≥ 2 µM. The pH-rate profiles for D104N and D206N variants were also determined at enzyme concentrations ≥ 2 µM using SctRNA^Phe^-G9 as substrate.

### 2.5. Metal Dependence Assays

Reactions containing the indicated labeled tRNAs, 50 mM Tris pH 8.0, 0.5 mM SAM, 3 µM enzyme and 125 µM–15 mM MgCl_2_ were incubated for 1 hour at 40 °C (SctRNAs) or 50 °C (TktRNAs). Since the maximum possible product varied for the different substrates with 3% (1/32 labeled G nucleotides) for uniformly labeled TktRNA^Arg^, 9% (1/11 labeled A nucleotides) for uniformly labeled TktRNA^Thr^ and 100% for the specifically R_9_-labeled SctRNAs, the observed percent product conversion (%*P*) in each assay was normalized for the maximal possible product for each substrate and plotted as a function of MgCl_2_ concentration. 

### 2.6. Differential Scanning Fluorimetry

In vitro transcribed full-length unlabeled tRNA (1 µg) was mixed with 400-fold diluted Quant-iT™ Ribogreen^®^ reagent (Life technologies, Carlsbad, CA, USA) and the fluorescence intensities and associated melting temperatures (*T*_m_) were measured on a CFX96 Real-Time PCR Detection system (Bio-Rad) using the in-system melting curve protocol [21].

### 2.7. Filter Binding Assay

tRNA binding assays were performed using filter-binding assay by incubating the respective uniformly-labeled tRNAs and 10 nM–10 µM enzyme in a reaction buffer containing 25 mM HEPES pH 7.5, 10 mM MgCl_2_, 3 mM DTT and 125 mM NaCl. The reactions were then filtered through a 96-well dot-blot apparatus with nitrocellulose and Hybond membranes to separate bound and unbound tRNA. The dried filters were exposed to a phosphor screen and the %bound tRNA were quantified using ImageQuant™ TL. The data were fit to Equation (3) (bound_min_ and bound_max_ are minimum and maximum limits of %bound tRNA, *n*–Hill coefficient, [E]–enzyme concentration) using Kaleidagraph (Synergy, Reading, PA, USA) to calculate *K*_D_.

%bound = bound_min_ + (bound_max_ − bound_min_)/(1 + (*K*_D_/[E])*^n^*) (3)

## 3. Results

### 3.1. pH-Rate Analysis to Assess the Mechanism of m^1^R_9_ Formation

Previous biochemical analysis of m^1^G_9_-specific enzymes ScTrm10 and hTRMT10A revealed a single catalytically-relevant ionization with a pKa of ~8. After ruling out the participation of several candidate ionizable side chains, the possibility that this pKa reflects the ionization of the N1 atom on the target G_9_ itself was considered [19]. Therefore, we reasoned that if bifunctional enzymes (catalyzing both m^1^A_9_ and m^1^G_9_) share the same rate-determining step for methylation as the monofunctional enzyme, these enzymes would also exhibit a different pattern of pH dependence for the two methylation reactions because of the difference in N1 pKa between adenine vs. guanine bases. Determining pH-rate profiles for the two activities catalyzed by the bifunctional methyltransferase TkTrm10 thus provided an opportunity to test this hypothesis. 

Single-turnover rates of m^1^G_9_ and m^1^A_9_ formation were determined for TkTrm10 using tRNA substrates uniquely-labeled with ^32^P immediately 5′ to the N_9_ nucleotide, as previously described [20], over a range of pH values between 5.0 and 9.0 (Figure 1B). To avoid sequence-dependent effects on catalysis outside of the target N_9_ residue, the tRNA substrates used were (1) the wild-type *S. cerevisiae* tRNA^Phe^ (SctRNA^Phe^), which naturally contains A_9_, and (2) a previously described variant of the same tRNA in which A_9_ has been replaced with G_9_ (SctRNA^Phe^-G9), thus rendering this tRNA a substrate for canonical m^1^G_9_ MTases, such as ScTrm10 [20]. These *S. cerevisiae* tRNA substrates were also readily methylated by TkTrm10 under our in vitro conditions, which included an assay temperature of 40 °C (slightly higher than the 30 °C typically used for the mesophile-derived SctRNAs and slightly lower than the 60 °C used previously for the thermophile-derived TkTrm10 enzyme [9,19]). 

With SctRNA^Phe^-G9, the pH dependence of the m^1^G_9_ formation by TkTrm10 was nearly identical to that observed for ScTrm10 and hTRMT10A, fit best by a single basic ionization with a pKa of 7.3 ± 0.1 (Figure 1B, filled squares). In contrast, the SctRNA^Phe^ m^1^A_9_ rates exhibited no dependence on pH across the tested range (Figure 1B, filled circles), indicating no catalytically relevant ionization in the enzyme-substrate complex occurring under these conditions. These data further support the previously proposed mechanism for both ScTrm10 and TkTrm10 in which the critical ionization observed during m^1^G_9_ catalysis corresponds to deprotonation of N1, with neither enzyme employing an essential general base residue for this reaction [9,19]. 

**Scheme 1:**
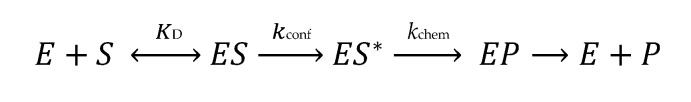
Minimal kinetic scheme for methylation by Trm10.

In each case, the measured single turnover *k*_obs_ were demonstrated to be independent of enzyme concentration, indicating that these represent the rate-determining step for chemistry and are not dependent on the formation of the ES complex. Hence, in the simplest kinetic scheme, this maximal rate reflects either the actual chemical step of methylation (*k*_chem_) or a conformational change that precedes the chemical step (*k*_conf_) (Scheme 1). In addition, we noted that the maximal overall rate of catalysis of m^1^G_9_ (1.39 ± 0.13 min^−1^) is about two-fold higher than the rate of m^1^A_9_ (0.56 ± 0.11 min^−1^) methylation even within the context of an otherwise identical tRNA backbone, suggesting that these two target nucleotides may be recognized in distinct ways within the enzyme active site. 

To further determine whether the tRNA sequence outside of R_9_ affects the catalytic properties of TkTrm10, pH-dependence was measured for three additional tRNA substrates, including two *T. kodakarensis* tRNA (TktRNA^Thr^ (m^1^A_9_), TktRNA^Arg^ (m^1^G_9_)) and one *S. cerevisiae* tRNA that has been studied extensively with eukaryotic Trm10 (SctRNA^Gly^ (m^1^G_9_)). To better compare the kinetic results for tRNA species of different origin, an assay temperature of 50 °C was used for TktRNA, slightly lower than the 60 °C used for the previous biochemical characterization [9], but closer to the 40 °C used for SctRNA in this work (Table 1). For SctRNA^Gly^, the entire pH-rate profile was determined, revealing a nearly identical pK_a_ to the SctRNA^Phe^-G9 substrate, but reflecting absolute rates that were ~10-fold lower for this m^1^G_9_ reaction (Figure 1B, open squares). Interestingly, a similar pH-dependent pattern of activity was observed with the TktRNA substrates, with m^1^G_9_ formation increasing across the pH range from 6.0–8.0, while m^1^A_9_ formation occurred at similar rates across this same range (Figure S2), but again with a substantial variation in observed rates exhibited for the two substrates. A comparison of measured *k*_obs_ for all five tested tRNA at pH 8.0 (at or near maximal activity for each substrate) revealed differences of up to ~100-fold between the various tRNA that do not obviously correlate with the identity of the nucleotide that is methylated, or with the species of origin for the tested tRNA (Table 1). The relatively low m^1^G_9_ rate observed with the TktRNA^Arg^ substrate (≤0.012 min^−1^) cannot be simply attributed to the hyperthermophilic origin of this tRNA relative to the assay conditions (50 °C), since the *k*_obs_ for the other *T. kodakarensis*-derived tRNA (TktRNA^Thr^) was ~10-fold higher and similar to that measured with SctRNA^Gly^. The significant tRNA-dependent rate variation observed in these assays is therefore most consistent with a rate-determining step comprising a conformational change (*k*_conf_), whose rate depends significantly on features associated with each substrate tRNA. 

### 3.2. Effect of Metals and tRNA Structural Stability on TkTrm10 Catalysis

**Figure 2:**
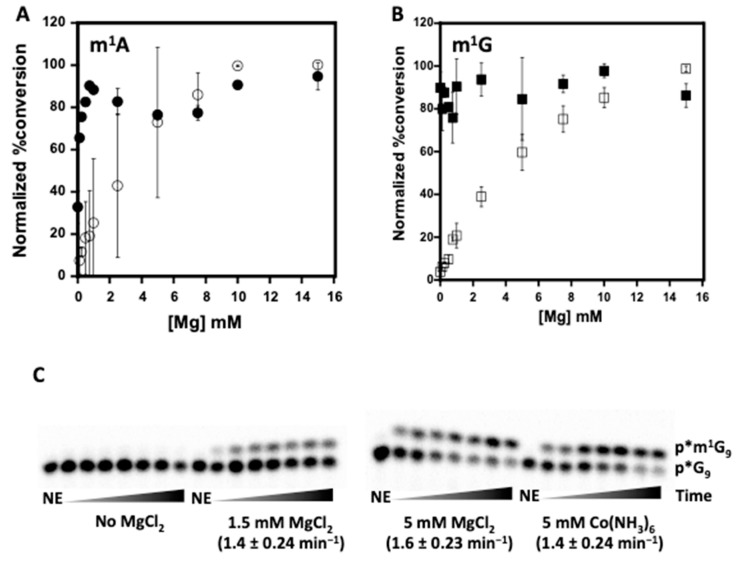
Substrate-dependent effects of Mg^2+^ on catalysis by TkTrm10. (**A**) Mg^2+^ dependence of m^1^A_9_ formation by TkTrm10 purified under metal-free conditions. Product formation after 1 h of reaction was quantified as described in methods for SctRNA^Phe^ (open circles) and TktRNA^Thr^ (filled circles). Each data point represents the average of at least two independent experiments, with error bars indicating standard deviation. (**B**) Mg^2+^ dependence of m^1^G_9_ formation by TkTrm10 purified under metal-free conditions. Product formation after 1 h of reaction was quantified as described in methods for SctRNA^Phe^-G9 (open squares) and TktRNA^Arg^ (filled squares). Each data point represents the average of at least two independent experiments, with error bars indicating standard deviation. (**C**) Time courses for m^1^G_9_ methylation of SctRNA^Phe^-G9 catalyzed by TkTrm10 in varied concentrations of added MgCl_2_ or Co(NH_3_)_6_, as indicated. Single turnover *k*_obs_ were determined by fit to Equation (1) and are reported in parentheses under the time course corresponding to each position.

A requirement for a divalent metal for m^1^G catalysis is a variable feature among SPOUT family MTases, with the m^1^G_37_-catalyzing TrmD requiring an Mg^2+^ ion to stabilize a negatively charged intermediate [22] while ScTrm10 shows no such requirement [19]. To test this for TkTrm10, we purified the enzyme without any added metal in the purification or storage buffers and tested activity on the same four substrates tested above, in the presence of increasing concentrations of Mg^2+^. Interestingly, the measured activity also varied in an unexpected tRNA substrate-dependent manner. The two tRNAs from *S. cerevisiae* required a much higher Mg^2+^ concentration (~6–10 mM in the assay) for maximal TkTrm10 activity compared to the two *T. kodakarensis* tRNAs, for which maximal activity was observed at ~1 mM Mg^2+^ or less (Figure 2A,B). Similar trends were exhibited for both m^1^G_9_ and m^1^A_9_ reactions, indicating that the identity of the target purine does not affect the observed metal dependencies. 

Since divalent metal ions are known to play critical roles in folding and overall structural stability of tRNA, we questioned whether the difference in Mg^2+^-dependence is the result of metal-dependent changes in affinity of TkTrm10 for certain tRNA. To test this, *K*_D_ for the same tRNA species were determined in the presence and absence of saturating Mg^2+^ (10 mM) using a double-filter binding assay. Interestingly, a modest (~2–10-fold) higher affinity for tRNA was observed for all tRNAs, regardless of species of origin, in the absence of added Mg^2+^ (Figure S3A,B). Thus, Mg^2+^-dependent differences in binding behavior do not explain the stronger dependence on added Mg^2+^ for *S. cerevisiae* tRNA. To further study this effect, we measured the single turnover rate of m^1^G_9_ formation for SctRNA^Phe^-G9 with no Mg^2+^, 1.5 mM Mg^2+^, 5 mM Mg^2+^ and 5 mM Co(NH_3_)_6_ (an exchange-inert metal complex that can functionally replace structural metal ions) (Figure 2C). Although no activity was detected in the absence of metal, activity was rescued with Mg^2+^, as well as by the exchange-inert cobalt hexamine, effectively ruling out inner-sphere coordination between the metal and substrate that is required for catalysis. Moreover, the measured *k*_obs_ at 1.5 mM and 5 mM Mg^2+^ were nearly identical, albeit with lower maximal percent conversion of the substrate in the presence of low concentrations of Mg^2+^ (likely reflecting an increased fraction of substrate that is competent for catalysis at higher Mg^2+^ concentrations) (Figure 2C). 

Since the rescue by cobalt hexamine suggested a structural role for metal ions, we compared determined the melting temperature (*T*m) for SctRNA^Phe^ and TktRNA^Thr^ using differential scanning fluorimetry (DSF) to compare their overall thermal stabilities. In this assay, RNA unfolding is monitored by binding to RiboGreen dye, which binds preferentially to single-stranded regions of RNA, thus, increasing fluorescence as the unfolding of secondary structures occurs as the temperature is increased [21]. A single apparently cooperative transition was observed for both tRNAs, but the TktRNA^Thr^ exhibited an increased *T*m of 65 °C compared to that of SctRNA^Phe^ (47.5 °C) for this unfolding event (Figure S3C), consistent with the hyperthermophilic origin of the *T. kodakarensis* tRNA. Thus, the more significant dependence on added Mg^2+^ for SctRNA^Phe^ correlates with its lower thermal stability, possibly indicating that metals are needed for some substrates to stabilize specific tRNA structural features associated with optimal catalysis. 

### 3.3. Mutational Analysis of Conserved Residues

**Figure 3:**
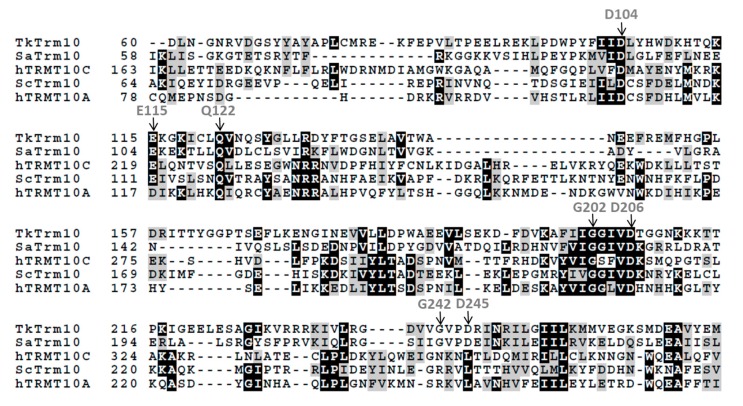
**Multiple sequence alignment of Trm10 homologs:** Alignment of eukaryotic (m^1^G_9_-specific ScTrm10 and hTRMT10A, m^1^R_9_-specific hTRMT10C) and archaeal (m^1^A_9_-specific SaTrm10, m^1^R_9_-specific TkTrm10) Trm10 homologs was performed using Clustal Omega [23]. The poorly conserved N-terminal domains of each enzyme are omitted for clarity, with the first residue number shown for each enzyme indicated to the left of the alignment. Conserved residues are highlighted by shading (using BoxShade, ExPASy, Lausanne, Switzerland), with the positions of residues analyzed in this study indicated by arrows above the alignment, using TkTrm10 numbering.

**Figure 4:**
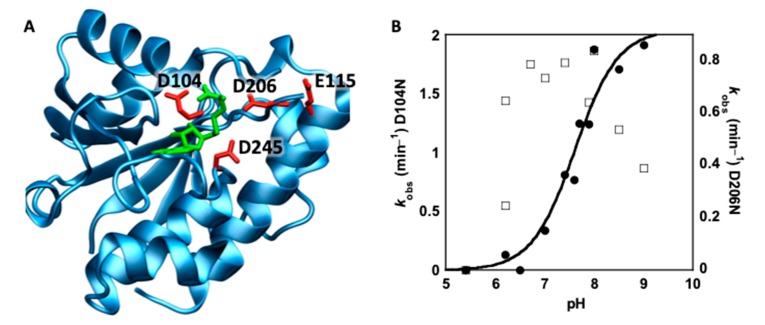
Analysis of conserved active site carboxylates. (**A**) Crystal structure of *T. kodakarensis* Trm10 (PDB: 6EMV) [9] showing the positions of D104, E115, D206 and D245. The *S*-adenosyl homocysteine is shown in green. (**B**) Single turnover *k*_obs_ (min^−1^) of m^1^G_9_ formation by D104N and D206N variants was measured at saturating enzyme concentrations. Rates of m^1^G_9_ formation in SctRNA^Phe^-G9 (from three independent experiments) were fit to Equation (2) to obtain the pKa values of 7.6 ± 0.1 for D104N (solid line, filled circles). A fit could not be determined for D206N (dashed line, open squares), due to very low rates below pH ~6.0. Note the difference in the *Y*-axis scale for comparison of the two variants.

Although the precise molecular mechanism used by Trm10 family enzymes is not known, several possible mechanisms have been evaluated by alteration of conserved putative active site residues. An early suggestion of general-base mediated catalysis by a conserved carboxylate was ruled out in the context of *S. cerevisiae* Trm10 by a combination of more sensitive kinetic assays and pH-rate profile analysis of variants [19]. More recently, minimal effects on catalysis upon alteration of two possible general-base carboxylates (D206 and D245) in the context of TkTrm10 revealed that methylation without the participation of an obligate general base is also likely a conserved feature among the dual function Trm10 enzymes [9]. The study showed that mutating either D206 or D245 to N or A only modestly decreases the catalytic rate of m^1^R_9_ formation and the rate is significantly impaired only in a D206A/D245A double mutant. Interestingly, however, these studies were performed with a different *T. kodakarensis* tRNA^Asp^ substrate (engineered like the SctRNA^Phe^ transcript here to be assayed for either m^1^G or m^1^A catalysis), for which even lower steady-state *k*_cat_ values (~10^−3^ min^−1^) were observed in the assays. Given our observation of significant tRNA substrate-dependent variation associated with TkTrm10 activity (Table 1), and the lack of information about two other conserved carboxylates that had been tested with ScTrm10, but not TkTrm10 (Figure 3, Figure 4A), we chose to look more deeply at the roles of putative active site residues and to determine whether previous trends also hold for the broader set of TkTrm10 substrates. 

First, to demonstrate that the single turnover kinetic assays are sensitive enough to detect the full range of effects on activity, due to active site alterations, we constructed and assayed a G202R TkTrm10 variant. G202 is a conserved glycine that forms part of the SAM-binding motif and interacts with the ribose moiety of SAH in the co-factor bound crystal structures of eukaryotic and archaeal homologs, including TkTrm10 (Figure 3) [7,8,9]. Using the high activity SctRNA^Phe^-derived substrates, we readily observed the expected nearly complete loss of both m^1^G_9_ and m^1^A_9_ activity associated with G202R TkTrm10, similar to ScTrm10 and human TRMT10A (Figure S4). The relatively poor yield of purified G202R enzyme (evident from the lower levels of this purified variant relative to co-purifying contaminants visible upon SDS-PAGE analysis, see Figure S1) suggests that there may also be some effect on TkTrm10 structural stability as a consequence of the G202R alteration. A control glycine residue that is conserved in archaeal Trm10 (G242), but is not predicted to participate in SAM binding according to the available structures was similarly altered; this G242R variant protein also purifies with similar levels of contaminants to G202R (Figure S1), but was readily active in the same assays (Figure S4). Therefore, G202 is important for catalysis by TkTrm10, regardless of the target purine nucleotide or tRNA species to be modified, and the in vitro assays can detect the effects of completely inactivating alterations.

Next, we constructed variants at the two carboxylates (D206 and D245) that were previously tested in the context of TkTrm10 with the low activity substrate TktRNA^Asp^, as well as two additional conserved residues (D104 and E115) that had been evaluated for catalytic roles in the context of ScTrm10, but not for any bifunctional Trm10 enzyme (Figure 3, Figure 4A) [9,19]. Single turnover rates (*k*_obs_) for methylation of the high activity SctRNA^Phe^ substrate were determined for the corresponding carboxamide versions of each side chain (D or E to either N or Q, respectively). Consistent with all previous observations for ScTrm10 and TkTrm10, none of the conserved carboxylates is strictly required for either m^1^A_9_ or m^1^G_9_ catalysis with the corresponding high activity tRNA^Phe^ substrates, based on the relatively modest effects on catalysis observed with each variant (Table 2). However, a comparison with previous results for D206N and D245N variants suggest some substrate-dependent effects of these residues on target nucleotide preference. With the archaeal tRNA^Asp^ substrate tested previously [9], wild-type TkTrm10 exhibited a modest preference in k_cat_ for A_9_ methylation (7.8 × 10^−3^ min^−1^ vs. 3.9 × 10^−3^ min^−1^ for G_9_) [9], while a slight opposite preference for G_9_ activity over A_9_ methylation was observed here for SctRNA^Phe^ (Table 2). Moreover, while the D245N alteration previously had no significant effect on the A-preference for TktRNA^Asp^ [9], this same alteration tested here exhibited a modest, but significant, reversal in nucleotide preference caused largely by a significant drop in activity on G_9_ (~4-fold) with the SctRNA^Phe^ substrate.

In ScTrm10, alteration of two conserved carboxylates (D100 and D210, equivalent to D104 and D206 in TkTrm10) caused an intriguing ~1 pH unit acid-shift in the observed pKa for m^1^G_9_ methylation. Here, we tested whether there was a similar effect on for m^1^G_9_ activities (on SctRNA^Phe^-G9) of D104N and D206N TkTrm10 that could reflect conserved mechanistic features. Interestingly, a markedly different pattern of pH-dependent behavior was observed for each of these variants in the context of TkTrm10. For D104N TkTrm10, a single pKa of 7.6 ± 0.1 was calculated, similar to wild-type TkTrm10 (Figure 4B, filled circles). On the other hand, the pH dependence of D206N TkTrm10 variant was significantly different. Although the very slow rates (and thus substantial error) associated with this variant made it difficult to measure precise *k*_obs_ at low pH (6.0 and lower), the rates were relatively pH-independent across the pH range ~6.0–8.0, suggesting a similar acid-shift in the pKa as observed with this same variant in the context of ScTrm10 (Figure 4B, open squares). Additionally, a possible acidic ionization at higher pH may be occurring with D206N TkTrm10, although pH-dependent effects on enzyme stability cannot be ruled out as the cause of the observed decrease in activity at high pH (Figure 4B).

As with the m^1^G_9_-specific ScTrm10, none of the single absolutely conserved carboxylates appears to be critical on its own for methylation. A D206N/D245N double variant exhibited modest effects on catalysis, nearly identical to those observed previously for the same variant assayed with the TktRNA^Asp^ substrate [9]. Indeed, only when all four conserved residues were altered in the D104N/D206N/D245N/E115Q (3D+E) variant were the rates significantly impacted (by up to 56-fold for m^1^A_9_ activity). However, even this more substantially reduced *k*_obs_ for the 3D+E variant agrees well with the calculated rate expected if the observed rate for this enzyme reflects the sum of the additive effects of each individual alteration (Table 2). 

The variants with N/Q substitutions for the carboxylate side chains, although no longer capable of acid-base catalysis mediated by the altered residues, are still competent for participating in hydrogen bonding interactions. Thus, two alanine variants were created in which the side chain functional group of the D104 and D245 residues that were relatively unaffected in rate by asparagine alteration. Indeed, more substantial activity losses compared to the corresponding D104N or D245N alterations were associated with each tested alanine variant (Table 2). A triple variant in which the D104A and D245A variants were combined with E115Q was tested, and although the ~10^3^-fold activity defect associated with this variant was more substantial than for either alteration alone, again the observed effects were not very different from the predicted rate according to the non-interacting model where each variant contributes independently to catalysis (Table 2). Thus, although the conserved residues may contribute modestly to catalysis, their roles are not critical and there is no evidence for redundant functions for any of these side chains in the active site. A general pattern also emerged in which the fold-effects of these alterations were somewhat greater on the m^1^G_9_ vs. m^1^A_9_ rate, which again indicate modest differences in these residues’ interaction(s) with the two purine nucleotides. 

Conflicting results for the role of Q122, which is analogous to the fungal Q118 residue implicated in the formation of the target G_9_ binding pocket, have been observed. While the fungal Q118A variant was reported to be catalytically inactive, the Q122A alteration in TkTrm10 exhibited only slightly decreased (37%) methyltransferase activity and no obvious effect on the preference for A_9_ methylation with the low activity TktRNA^Asp^ substrate [7,9]. Here again, kinetic analysis with the high activity SctRNA^Phe^-derived substrates suggests additional substrate-dependent complexity, with a modest effect on m^1^A_9_ activity (~4-fold) that is similar to that observed when assayed with TktRNA^Asp^, but a much stronger decrease in m^1^G_9_ methylation (~42-fold) caused by this alteration (Table 2). Importantly, however, the Q residue is highly conserved even in family members (such as SaTrm10) that only act on A_9_, suggesting that its role is likely more complicated than simply binding to the target guanosine base. 

## 4. Discussion

Recent mechanistic and structural characterizations of Trm10 homologs have revealed that these enzymes comprise a catalytically distinct group of SPOUT-family methyltransferases, with several characteristics that distinguish them from the two other known tRNA m^1^G MTases, Trm5 and TrmD [7,9,19,24]. However, despite crystal structures that are now available for homologs exhibiting each type of target nucleotide specificity (G_9_ only, A_9_ only, or bifunctional G_9_/A_9_ enzymes), the basis of substrate nucleotide specificity remains poorly understood. In this work, we aimed to further evaluate the active site features of m^1^R_9_ dual-specific Trm10 enzymes through biochemical characterization of TkTrm10. 

Previous characterization of the m^1^A_58_ methyltransferase TrmI, a Class I Rossman-fold enzyme, suggested two possible catalytic mechanisms for m^1^A formation [25]. In both of these, an active site aspartate (D170) interacts with the α-NH_3_ group of SAM, and has been suggested to act either as: (i) A general base to abstract the N6 proton to form a deprotonated imino tautomer that promotes nucleophilicity of the N1 atom or (ii) a structural factor to bring the two substrates (SAM and A_58_) in the right orientation for methylation [25]. Although not rigorously excluded, a mechanism involving D170 as a general base to deprotonate N6 in the TrmI family has been disfavored for multiple reasons. First, the pKa of the N6 on unmodified adenine is significantly higher (>16.7) [26] than that of m^1^A N6 (9.6) [27] implying that its deprotonation is more likely to occur after, or at least simultaneously with, methylation [25]. Second, the free energy of methyl transfer from SAM to form SAH is relatively high, and methylation can even occur spontaneously when provided with a methyl acceptor in the right orientation [25,28,29]. Finally, mutation of D170 to N does not result in a complete loss of activity indicating a non-essential role for this residue as a general base [25,30]. Therefore, the mechanism in which D170 simply brings the two substrates in the correct orientation in close proximity has been favored [25,31]. 

Remarkably, despite belonging to two evolutionarily and structurally distinct MTase families, parallels can be drawn between the active sites of TrmI and Trm10. The aspartate D210 in ScTrm10 (D206 in TkTrm10), like D170 of TrmI, interacts with the α-NH_3_ group of SAH in the solved crystal structure and had been initially predicted to take on the role of a general base [7,18]. Moreover, as with D170 of TrmI, kinetic analysis of protein variants ruled out an essential general base role for the D206 residue in m^1^G_9_ or m^1^A_9_ catalysis in any tested enzyme (Table 2) [9,19]. In this current work, a definitive case against a general base requirement for m^1^A_9_ catalysis can be made based on the pH-rate analysis that showed no rate-determining ionization required for A_9_ methylation across a broad physiological pH range (Figure 1). These observations all favor a role for D206 where it may serve to bring the methyl-donor and R_9_ together in the right orientation for m^1^R_9_ catalysis, as has been suggested for D170 of TrmI. 

Based on the results of the kinetic analysis presented here, one plausible model for catalysis is that the conserved active site residues in Trm10 are involved in hydrogen bonding interactions to position substrates in the correct orientation for methyl transfer. This model is supported by our observations of significant substrate-tRNA dependent differences in single-turnover methylation rates (Table 1). These differences suggest that the rate-determining step for catalysis by TkTrm10 is not the chemistry itself, but a slower structural or conformational change associated with the ES complex, whose rate depends on specific features of the substrate tRNA (Scheme 1). Since this [enzyme]-independent rate constant is affected to the varying extent by altering the conserved active site carboxylates, we hypothesize that the tested carboxylate residues participate in a structural change that facilitates accommodation of R_9_ in the active site (possibly through a hydrogen bonding network). The catalytic model is further supported by the less severe catalytic defects that are generally exhibited by carboxamide-substituted (N or Q) variants compared to the variants where the same positions are altered to alanine (Table 2). 

Understanding the molecular basis for variable purine nucleotide specificities exhibited by various Trm10 family enzymes is an important goal, and this study also reveals some unappreciated complexity associated with this question. In the previous kinetic analysis of TkTrm10 with the TktRNA^Asp^ substrate, low *k*_cat_ values (~10^3^-fold lower than ScTrm10) were observed for both A_9_ and G_9_ methylation [9]. Although the intriguing possibility of additional protein cofactors required for optimal TkTrm10 activity (as observed for the human mitochondrial TRMT10C enzyme) was raised and these may yet be identified, our kinetic characterization of a number of tRNA substrates derived from both *S. cerevisiae* and *T. kodakarensis* reveals that the slow activities are not an inherent feature of the isolated enzyme, but that the *S. cerevisiae*-derived tRNAs are associated with relatively high single-turnover rates that are similar to those observed in assays with ScTrm10 [19]. Although the thermophilic character of the TktRNA may be somewhat incompatible with the temperature of the assays (50 °C) conducted here, the ~10-fold difference in rates even between the two TktRNAs suggests that there are additional factors at work. Indeed, in addition to substrate-dependent effects on overall rate, we demonstrate here that the patterns of catalytic preference for methylating G vs. A, effects of alterations at conserved residues, and metal-ion dependence can all differ depending on the substrate being assayed. Overall, a picture is emerging that implicates specific tRNA features in catalysis, and the preference for methylation at A_9_ or G_9_ may be simply a broader reflection of the ability of this nucleotide in the context of a specific tRNA to be positioned correctly in the active site. 

Here we have identified at least one substrate-dependent pattern that is consistent with this idea, by demonstrating that increased stability of the target tRNA (as judged by overall *T*m) correlates with increased product formation. This was inferred from the metal-dependence of methylation by TkTrm10, where the comparatively less stable SctRNA^Phe^ (*T*m = 47.5 °C) requires higher concentrations of metal ions (which presumably act to increase structural stability) in the reaction to achieve similar product conversion compared to the more thermostable TktRNAs (*T*m of TktRNA^Thr^ = 65 °C) (Figure 2A, Figure 2B, Figure S3C). Interestingly, this distinction could not be attributed to overall preferential binding of more stable tRNAs by TkTrm10, as all tRNAs bound with similar affinity under a given concentration of metal ions (Figure S3A,B). An intriguing possibility that could explain these observations is that TkTrm10 uses the three-dimensional structure of substrate tRNAs to locate R_9_, and therefore more stable tRNAs with intact folded structures result in higher fractions of catalytically competent ES complexes. This hypothesis is also consistent with a computational docking model of *E. coli* initiator tRNA with a crystal structure of full length SaTrm10 (another archaeal homolog), in which it was predicted that the overall tRNA-like shape of the enzyme complements the tertiary structure of the substrate tRNA [8]. 

As a group, the Trm10 enzymes have so far proven to comprise a distinct class of enzymes that employ catalytic features of both SPOUT and Class I MTases. The trefoil knot used to bind SAM is an obligate feature of SPOUT MTases, and the structural classification of Trm10 into this superfamily on this basis is unambiguous [7,8,32]. However, aside from this SAM binding motif, few biochemical characteristics have been in line with other features typically associated with the SPOUT family. This includes the apparent monomeric form of Trm10, which is more like that of the Class I m^1^G MTase Trm5 [33,34]. Moreover, based on the current tRNA-binding model, Trm10 may recognize the entire L-shaped structure of the tRNA [8]; this is also a catalytic feature associated with Trm5, which performs a tertiary fold check by interacting with the D-loop before catalyzing the anticodon modification [35]. Finally, the catalytic mechanism of m^1^A_9_ catalysis shares some obvious features with that used by the Class I MTase TrmI to catalyze m^1^A_58_ modification. Further studies of the basis for tRNA recognition and base specificity of Trm10 therefore could address the possibility of Trm10 forming a distinct MTase family that is characterized by specific features borrowed from both SPOUT and Class I enzymes. 

## Figures and Tables

**Table 1:** Substrate dependent differences in single turnover methylation rate by TkTrm10.

Substrate	*k*_obs_ min^−1^ (pH 8.0)	Assay Temperature
**m^1^G_9_**		
SctRNA^Phe^-G9	1.39 ± 0.13	40 °C
SctRNA^Gly^	0.15 ± 0.04	40 °C
TktRNA^Arg^	≤0.012 *^a^*	50 °C
**m^1^A_9_**		
SctRNA^Phe^	0.56 ± 0.11	40 °C
TktRNA^Thr^	0.12 ± 0.02	50 °C

*^a^* Upper limit to *k*_obs_ derived using method of initial rates.

**Table 2:** Single turnover rates of m^1^A_9_ and m^1^G_9_ methylation by TkTrm10 variant enzymes.

TkTrm10 Variant	m^1^A_9_ *k*_obs_ (min^−1^)	Theoretical *k*_obs_ (min^−1^) *^c^*	m^1^A_9_ FD *^a^*	m^1^G_9_ *k*_obs_ (min^−1^)	Theoretical *k*_obs_ (min^−1^)*^c^*	m^1^G_9_ FD *^a^*
WT	0.56 ± 0.11	-	-	1.4 ± 0.13	-	-
D104N	0.49 ± 0.08	-	1.2	1.74 ± 0.20	-	0.8
E115Q	0.38 ± 0.06	-	1.5	0.42 ± 0.07	-	3.3
D206N	0.17 ± 0.04	-	3.4	0.57 ± 0.10	-	2.4
D245N	0.40 ± 0.06	-	1.4	0.34 ± 0.01	-	4.0
D206N+D245N	0.14 ± 0.03	0.12	4.0	0.23 ± 0.01	0.14	6.0
D104N+D206N+D245N (3D)	0.09 ± 0.01	0.10	6.0	0.32 ± 0.06	0.17	4.4
D104N+D206N+D245N+E115Q (3D+E)	0.013 ± 0.007	0.07	56	0.13 ± 0.01	0.05	11
D104A	0.035 ± 0.007	-	16	0.071 ± 0.003	-	20
D245A	0.021 ± 0.008	-	26	0.021 ± 0.008	-	68
D104A+E115Q+D245A (DED)	<0.0006 *^b^*	0.0009	1555	<0.0006 *^b^*	0.0004	3487
Q122A	0.14 ± 0.02	-	4.0	0.03 ± 0.01	-	42

***^a^*** FD—fold defect for the indicated variant, calculated by comparison to WT *k*_obs_. *^b^* Upper limit to *k*_obs_ calculated using the method of linear initial rates. ***^c^*** Theoretical *k*_obs_ value calculated assuming the effects of multiple alterations on catalysis are additive, according to Reference [19].

## Supplementary Materials

The following are available online at http://www.mdpi.com/2073-4425/10/2/100/s1, Figure S1: Purified TkTrm10 proteins, Figure S2: pH dependence trends of m^1^G_9_ and m^1^A_9_ formation by TkTrm10 in native substrates, Figure S3: [Mg^2+^] dependence of TkTrm10-tRNA binding, Figure S4: Both purine methylation activities of TkTrm10 utilize the same SAM binding site, Figure S5: Location of the TkTrm10 target R_9_ in the tRNA core.

## References

[1] Jackman J.E., Alfonzo J.D. (2013). Transfer RNA modifications: Nature’s combinatorial chemistry playground. Wiley Interdiscip. Rev. RNA.

[2] Hori H. (2014). Methylated nucleosides in tRNA and tRNA methyltransferases. Front. Genet..

[3] Motorin Y., Helm M. (2011). RNA nucleotide methylation. Wiley Interdiscip. Rev. RNA.

[4] Hou Y.-M., Perona J.J. (2010). Stereochemical mechanisms of tRNA methyltransferases. FEBS Lett..

[5] Krishnamohan A., Jackman J.E. (2018). A Family Divided: Distinct Structural and Mechanistic Features of the SpoU-TrmD (SPOUT) Methyltransferase Superfamily. Biochemistry.

[6] Jackman J.E., Montange R.K., Malik H.S., Phizicky E.M. (2003). Identification of the yeast gene encoding the tRNA m^1^G methyltransferase responsible for modification at position 9. RNA.

[7] Shao Z., Yan W., Peng J., Zuo X., Zou Y., Li F., Gong D., Ma R., Wu J., Shi Y. (2014). Crystal structure of tRNA m^1^G9 methyltransferase Trm10: insight into the catalytic mechanism and recognition of tRNA substrate. Nucleic Acids Res..

[8] Van Laer B., Roovers M., Wauters L., Kasprzak J.M., Dyzma M., Deyaert E., Kumar Singh R., Feller A., Bujnicki J.M., Droogmans L. (2016). Structural and functional insights into tRNA binding and adenosine N1-methylation by an archaeal Trm10 homologue. Nucleic Acids Res..

[9] Singh R.K., Feller A., Roovers M., Elder D.V.A.N., Wauters L., Droogmans L., Versées W. (2018). Structural and biochemical analysis of the dual-specificity Trm10 enzyme from Thermococcus kodakaraensis prompts reconsideration of its catalytic mechanism. RNA.

[10] Helm M., Giegé R., Florentz C. (1999). A Watson-Crick base-pair-disrupting methyl group (m1A9) is sufficient for cloverleaf folding of human mitochondrial tRNALys. Biochemistry.

[11] Gillis D., Krishnamohan A., Yaacov B., Shaag A., Jackman J.E., Elpeleg O. (2014). TRMT10A dysfunction is associated with abnormalities in glucose homeostasis, short stature and microcephaly. J. Med. Genet..

[12] Zung A., Kori M., Burundukov E., Ben-Yosef T., Tatoor Y., Granot E. (2015). Homozygous deletion of TRMT10A as part of a contiguous gene deletion in a syndrome of failure to thrive, delayed puberty, intellectual disability and diabetes mellitus. Am. J. Med. Genet. A.

[13] Igoillo-Esteve M., Genin A., Lambert N., Désir J., Pirson I., Abdulkarim B., Simonis N., Drielsma A., Marselli L., Marchetti P. (2013). tRNA methyltransferase homolog gene TRMT10A mutation in young onset diabetes and primary microcephaly in humans. PLoS Genet..

[14] Yew T.W., McCreight L., Colclough K., Ellard S., Pearson E.R. (2015). tRNA methyltransferase homologue gene TRMT10A mutation in young adult-onset diabetes with intellectual disability, microcephaly and epilepsy. Diabet. Med..

[15] Metodiev M.D., Thompson K., Alston C.L., Morris A.A.M., Bahi-buisson N., Pyle A., Griffin H., He L., Assouline Z., Siira S. (2016). Recessive Mutations in TRMT10C Cause Defects in Mitochondrial RNA Processing and Multiple Respiratory Chain Deficiencies. Am. J. Hum. Genet..

[16] Gustavsson M., Ronne H. (2008). Evidence that tRNA modifying enzymes are important in vivo targets for 5-fluorouracil in yeast. RNA.

[17] Kempenaers M., Roovers M., Oudjama Y., Tkaczuk K.L., Bujnicki J.M., Droogmans L. (2010). New archaeal methyltransferases forming 1-methyladenosine or 1-methyladenosine and 1-methylguanosine at position 9 of tRNA. Nucleic Acids Res..

[18] Vilardo E., Nachbagauer C., Buzet A., Taschner A., Holzmann J., Rossmanith W. (2012). A subcomplex of human mitochondrial RNase P is a bifunctional methyltransferase–extensive moonlighting in mitochondrial tRNA biogenesis. Nucleic Acids Res..

[19] Krishnamohan A., Jackman J.E. (2017). Mechanistic features of the atypical tRNA m^1^G9 SPOUT methyltransferase, Trm10. Nucleic Acids Res..

[20] Swinehart W.E., Henderson J.C., Jackman J.E. (2013). Unexpected expansion of tRNA substrate recognition by the yeast m^1^G9 methyltransferase Trm10. RNA.

[21] Silvers R., Keller H., Schwalbe H., Hengesbach M. (2015). Differential Scanning Fluorimetry for Monitoring RNA Stability. Chembiochem.

[22] Sakaguchi R., Lahoud G., Christian T., Gamper H., Hou Y.-M. (2014). A divalent metal ion-dependent N^1^-methyl transfer to G37-tRNA. Chem. Biol..

[23] Sievers F., Wilm A., Dineen D., Gibson T.J., Karplus K., Li W., Lopez R., Mcwilliam H., Remmert M., Sö Ding J. (2011). Fast, scalable generation of high-quality protein multiple sequence alignments using Clustal Omega. Mol. Syst. Biol..

[24] Swinehart W.E., Jackman J.E. (2015). Diversity in mechanism and function of tRNA methyltransferases. RNA Biol..

[25] Dégut C., Ponchon L., Folly-Klan M., Barraud P., Tisné C. (2016). The m1A58 modification in eubacterial tRNA: An overview of tRNA recognition and mechanism of catalysis by TrmI. Biophys. Chem..

[26] Stewart R., Harris M.G. (1977). Amino group acidity in nucleotide bases. Can. J. Chem..

[27] Kettani A., Guéron M., Leroy J.-L. (1997). Amino Proton Exchange Processes in Mononucleosides. J. Am. Chem. Soc..

[28] Schubert H.L., Blumenthal R.M., Cheng X. (2003). Many paths to methyltransfer: A chronicle of convergence. Trends Biochem. Sci..

[29] Rydberg B., Lindahl T. (1982). Nonenzymatic methylation of DNA by the intracellular methyl group donor S-adenosyl-L-methionine is a potentially mutagenic reaction. EMBO J..

[30] Barraud P., Golinelli-Pimpaneau B., Atmanene C., Sanglier S., Van Dorsselaer A., Droogmans L., Dardel F., Tisné C. (2008). Crystal Structure of *Thermus thermophilus* tRNA m^1^A_58_ Methyltransferase and Biophysical Characterization of Its Interaction with tRNA. J. Mol. Biol..

[31] Oerum S., Dégut C., Barraud P., Tisné C. (2017). m1A Post-Transcriptional Modification in tRNAs. Biomolecules.

[32] Tkaczuk K.L., Dunin-Horkawicz S., Purta E., Bujnicki J.M. (2007). Structural and evolutionary bioinformatics of the SPOUT superfamily of methyltransferases. BMC Bioinform..

[33] Christian T., Lahoud G., Liu C., Hoffmann K., Perona J.J., Hou Y.-M. (2010). Mechanism of N-methylation by the tRNA m1G37 methyltransferase Trm5. RNA.

[34] Christian T., Gamper H., Hou Y. (2013). Conservation of structure and mechanism by Trm5 enzymes. RNA.

[35] Goto-Ito S., Ito T., Kuratani M., Bessho Y., Yokoyama S. (2009). Tertiary structure checkpoint at anticodon loop modification in tRNA functional maturation. Nat. Struct. Mol. Biol..

