# Supplementary material for: Insights into Catalytic and tRNA Recognition Mechanism of the Dual-Specific tRNA Methyltransferase from Thermococcus kodakarensis
Source: Genes (Basel). 2019 Jan 30;10(2):100. doi: 10.3390/genes10020100 (PMC6410153; doi:10.3390/genes10020100)
Supplement: Supplementary file 1 [file genes-10-00100-s001.pdf]

__Supplemental Material for:__

Insights into catalytic and tRNA recognition mechanism of the dual-specific tRNA methyltransferase from *Thermococcus kodakarensis*

Aiswarya Krishnamohan[1], Samantha Dodbele[1] and Jane E. Jackman[1*]

Figure S1

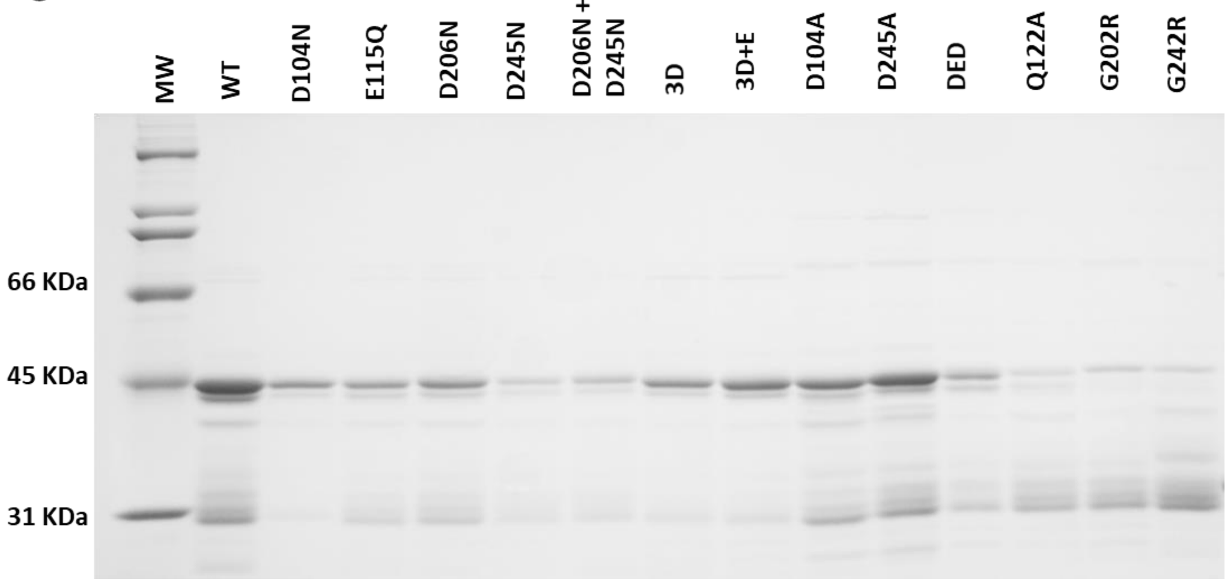

Figure S1: Purified TkTrm10 proteins. SDS-PAGE of purified TkTrm10 wild type and mutant proteins. Expected molecular weights for the N-terminal His_6-tagged enzymes is 44 kDa. MW – Molecular weight standards; WT – wild-type; 3D – D104N+D206N+D245N; 3D+E – D104N+D206N+D245N+E115Q; DED – D100A+E115Q+D245A

Figure S2

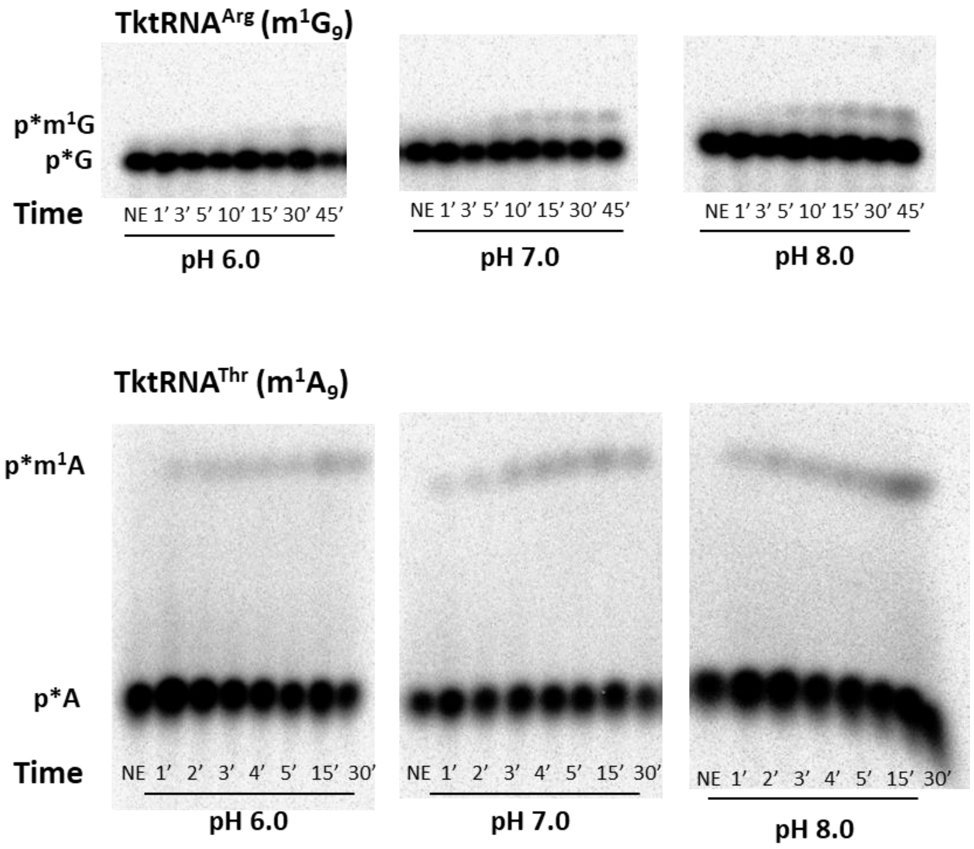

Figure S2: pH dependence trends of m^1G_9 and m^1A_9 formation by TkTrm10 in transcripts representing endogeonous *Thermococcus. kodakarensis* substrates: Time courses of m^1G_9 and m^1A_9 formation in TktRNA^{Arg} and TktRNA^{Thr} respectively at various pH conditions. m^1G_9 formation increases at higher pH conditions whereas m^1A_9 formation remains unaffected.

Figure S3

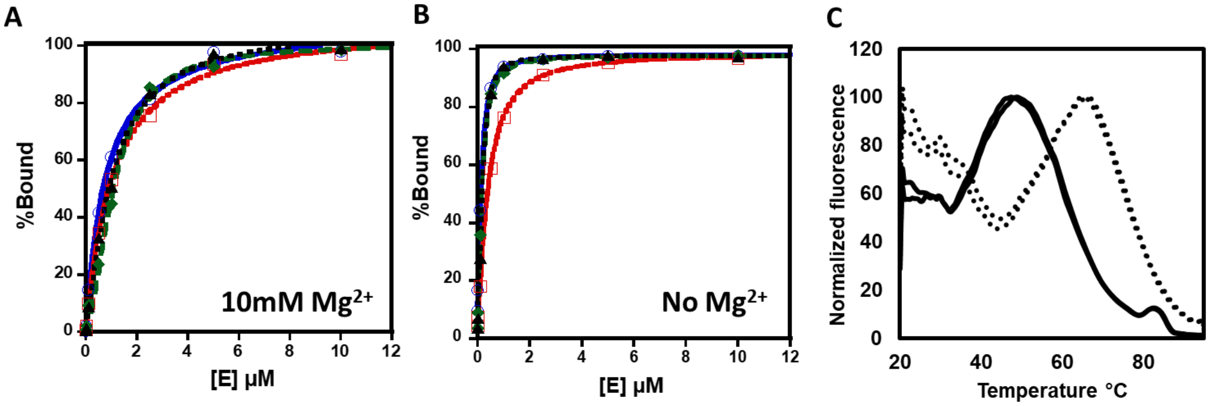

tRNA	$K_{D\,(tRNA)}$ µM (10mM Mg^{2+})	$K_{D\,(tRNA)}$ µM (No Mg^{2+})
TktRNA^{Thr}	0.79 ± 0.10 (0.53 – 0.79)	0.14 ± 0.01 (0.14 – 0.57)
TktRNA^{Arg}	1.04 ± 0.11 (0.68 – 2.8)	0.40 ± 0.01 (0.4 – 1.3)
SctRNA^{Phe}	1.11 ± 0.10 (0.46 – 1.1)	0.16 ± 0.09
SctRNA^{Phe}-G9	1.01 ± 0.10 (0.76 – 1.2)	0.18 ± 0.01 (0.18 – 0.65)

Figure S3: [Mg^{2+}] dependence of TkTrm10-tRNA binding: Filter binding assay was performed under conditions of either 10 mM Mg^{2+} **(A)** or no Mg^{2+} **(B)** with TkTrm10 and different uniformly labeled substrate tRNAs. The %bound tRNA was quantified and plotted as a function of TkTrm10 concentration, [E], and fit to Equation (5) to obtain the $K_{D(tRNA)}$. Representative fits from one experiment are shown with the range of $K_{D(tRNA)}$ measured from at least two independent experiments indicated in parentheses. (Blue = TktRNA^{Thr}; Red = TktRNA^{Arg}; Green = SctRNA^{Phe}; Black = SctRNA^{Phe}-G9) **(C)** Differential Scanning Fluorimetry of SctRNA^{Phe} and TktRNA^{Thr}. Since the binding efficiency of the Ribogreen dye decreases with increasing temperature, the first differential of the fluorescence was normalized to the maximum fluorescence at the peak of the first unfolding event and plotted as a function of time for direct comparison of the two tRNAs. The melting temperatures were estimated at 47.5°C and 65°C for SctRNA^{Phe} and TktRNA^{Thr} respectively from two independent experiments.

Figure S4

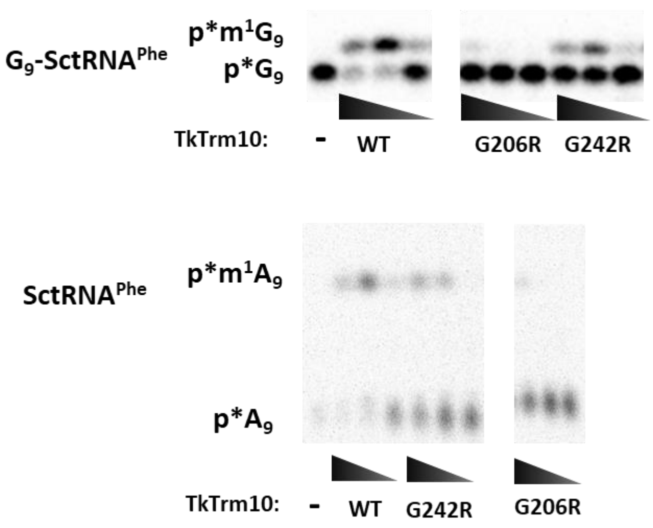

Figure S4: Both purine methylation activities of TkTrm10 utilize the same SAM binding site: *In vitro* methylation assays of m^{1}G$_9$ and m^{1}A$_9$ formation by TkTrm10 variants (WT, G202R and G242) with specific labeled G$_9$-SctRNA^{Phe} and SctRNA^{Phe} respectively. Reactions contain 10-fold serial dilutions of the respective variant or no enzyme (-). The G202R variant targeting the presumed TkTrm10 SAM-binding motif results in a significant loss of both methylation activities compared to G242R at a different location on the protein.

Figure S5

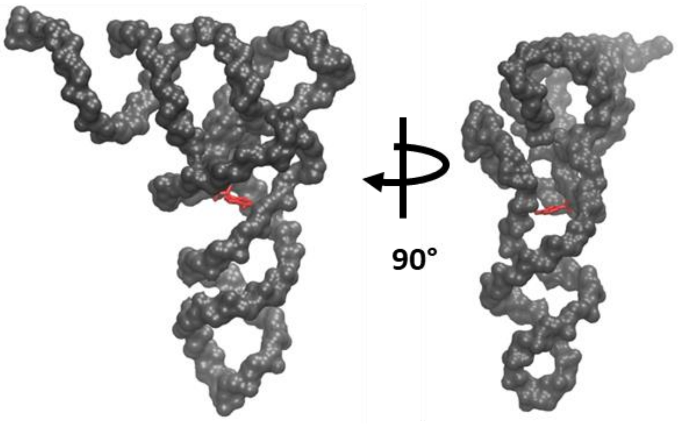

Figure S5: Location of the TkTrm10 target R$_9$ in the tRNA core. Crystal structure of SctRNA^{Phe} (1EHZ) with the position of adenosine at position 9 (in red) in the tRNA core.
